# Assessing the Accuracy and Efficiency of Free Energy Differences Obtained from *Reweighted* Flow-Based Probabilistic Generative Models

**DOI:** 10.1021/acs.jctc.4c00520

**Published:** 2024-07-10

**Authors:** Edgar Olehnovics, Yifei Michelle Liu, Nada Mehio, Ahmad Y. Sheikh, Michael R. Shirts, Matteo Salvalaglio

**Affiliations:** † Thomas Young Centre and Department of Chemical Engineering, University College London, London WC1E 7JE, U.K.; ‡ Molecular Profiling and Drug Delivery, Research & Development, AbbVie Bioresearch Center, Worcester, Massachusetts 01605, United States; § Molecular Profiling and Drug Delivery, Research & Development, AbbVie Inc, North Chicago, Illinois 60064, United States; ∥ University of Colorado Boulder, Boulder, Colorado 80309, United States

## Abstract

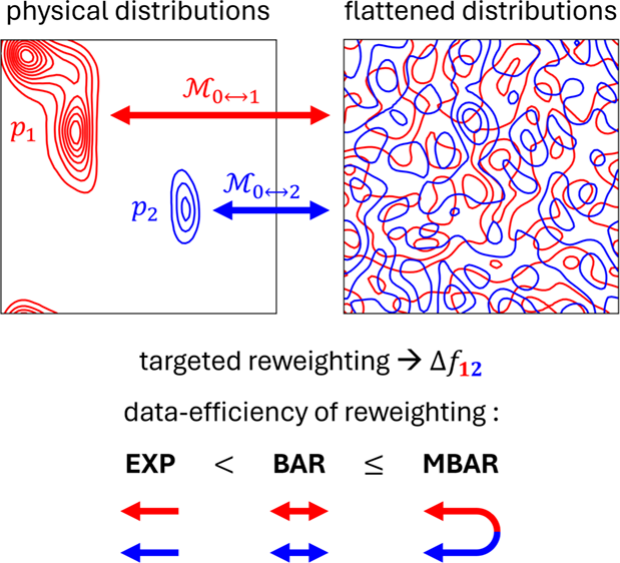

Computing free energy differences between metastable states characterized by nonoverlapping Boltzmann distributions is often a computationally intensive endeavor, usually requiring chains of intermediate states to connect them. Targeted free energy perturbation (TFEP) can significantly lower the computational cost of FEP calculations by choosing a set of invertible maps used to directly connect the distributions of interest, achieving the necessary statistically significant overlaps without sampling any intermediate states. Probabilistic generative models (PGMs) based on normalizing flow architectures can make it much easier via machine learning to train invertible maps needed for TFEP. However, the accuracy and applicability of approaches based on empirically learned maps depend crucially on the choice of reweighting method adopted to estimate the free energy differences. In this work, we assess the accuracy, rate of convergence, and data efficiency of different free energy estimators, including exponential averaging, Bennett acceptance ratio (BAR), and multistate Bennett acceptance ratio (MBAR), in reweighting PGMs trained by maximum likelihood on limited amounts of molecular dynamics data sampled only from end-states of interest. We carry out the comparisons on a set of simple but representative case studies, including conformational ensembles of alanine dipeptide and ibuprofen. Our results indicate that BAR and MBAR are both data efficient and robust, even in the presence of significant model overfitting in the generation of invertible maps. This analysis can serve as a stepping stone for the deployment of efficient and quantitatively accurate ML-based free energy calculation methods in complex systems.

## 1 Introduction

Physics-based modeling plays a dominant role in computational material design, enabling the prediction of physical properties of molecular materials and guiding their synthesis. Estimating the thermodynamic stability of molecular systems with efficient computational methods would drastically improve the utility of physics-based models at every stage of the design and development of organic crystalline materials, including therapeutics,^1,2^ from assessing the potency of a drug candidate to evaluating its crystallizability in an effective form.

Traditional molecular modeling and simulation methods based on statistical mechanics principles provide accurate but computationally expensive ways of estimating thermodynamic stability by calculating free energy differences between metastable states of interest.^3,4^ Free energy perturbation (FEP) methods are a representative and general class of free energy methods that are currently applied across all domains of computational physical chemistry, from the calculation of binding affinities to the estimate of crystalline lattice free energies.^5−10^ FEP methods aim to empirically estimate the ratios of partition functions *Z*_*i*_/*Z*_*j*_, using finite sets of samples {***r***}, sampled via simulations of states *i* and *j*, thus yielding (temperature-reduced) free energy differences Δ*f*_*ij*_ = *f*_*i*_–*f*_*j*_ = −ln (*Z*_*i*_/*Z*_*j*_). In the canonical ensemble, these distributions have the following native form *p*_*i*_ = *p*_*i*_(***r***) = *Z*_*i*_^–1^ exp (−*u*_*i*_(***r***)), where ***r*** are Cartesian coordinates of all atoms, and *u*_*i*_ = (*k*_B_*T*)^−1^*U*_*i*_ are different reduced potential energy functions (in temperature-reduced units of *k*_B_*T*). The normalization constants of these distributions *Z*_*i*_ and *Z*_*j*_, i.e., their partition functions, are typically unknown a priori.

In the presence of statistically significant overlaps between the sampled distributions of states *i* and *j*, reweighting methods, such as exponential averaging (EXP),^11^ Bennett acceptance ratio (BAR),^12^ and multistate Bennett acceptance ratio (MBAR),^13,14^ can be directly applied to estimate Δ*f*_*ij*_.

When states *i* and *j* are characterized by nonoverlapping or poorly overlapping distributions, instead, a typical classical approach followed in FEP-based methods is to sample a *chain* of additional Boltzmann distributions {*p*_1_,..., *p*_*K*_} joining the end-states of interest. Such distributions are obtained performing simulations in states with locally overlapping distributions that empirically trace a path between end states *i* = 1 and *j* = *K* in physical or alchemical space.

While rigorous and conceptually intuitive, collecting sufficient numbers of samples along such paths to converge the end point free energy distributions can be computationally demanding. In many cases, most of the computational efforts are spent sampling the intermediate states that may hold no inherent physical meaning.

Fortunately, this computational resource overhead can be partially or entirely minimized when considering diverse scenarios in which it is possible to obtain an accurate enough approximation of a deterministic bijective coordinate transformation directly mapping between states *i* and *j* without the need for sampling any intermediate states.

This idea is at the heart of so-called *targeted* reweighting methods, such as targeted EXP,^15^ targeted BAR,^16^ and targeted MBAR,^17^ which can yield accurate Δ*f*_*ij*_ estimates even in the absence of any explicit overlaps between the configurational ensembles of data sets sampled in states *i* and *j*.

**Figure 1:**
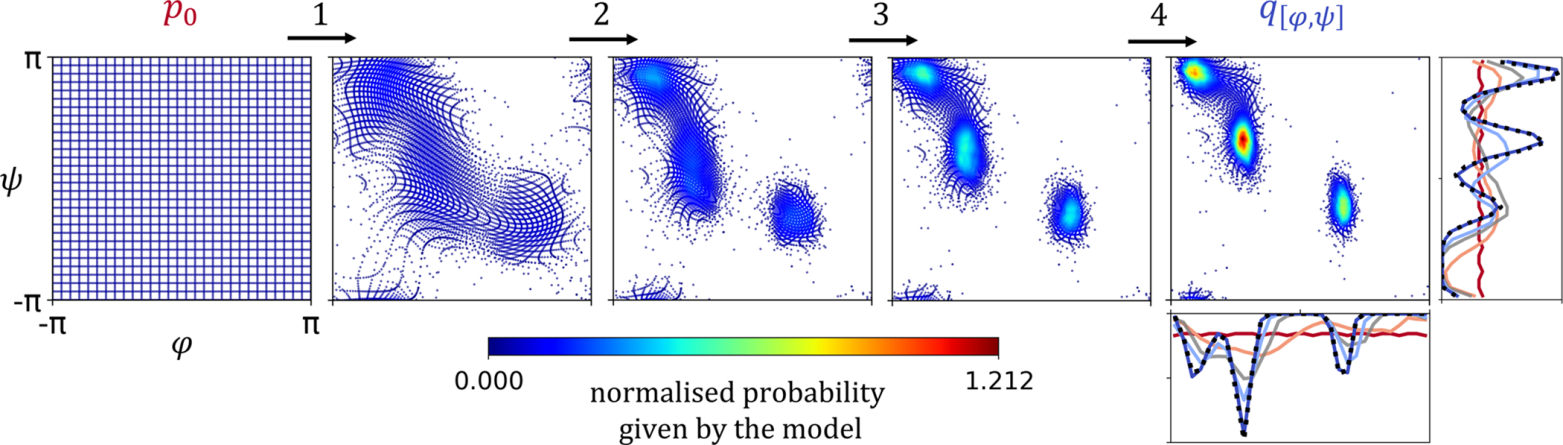
Illustration of spline coupling layers transforming uniform base distribution to approximate the target distribution in a 2D scenario. The uniform base distribution *p*_0_ (red) is transformed by discrete amounts via a sequence of four coupling layers (numbered arrows) to give *q*_[ϕ,ψ]_ (blue), which approximates the target distribution *p*_[ϕ,ψ]_. The marginals of the target distribution are shown using histograms (dashed black curves). After each coupling layer, normalized probabilities of the transformed samples can be evaluated (color bar). The same is true in reverse (not shown).

Similar to prior literature,^18−21^ in this paper, we separately train *K* independent flow-based probabilistic generative models (PGMs), to approximate invertible coordinate transformations (i.e., maps) between the physical ensembles we care about (*p*_*i*_; *i* = 1,..., *K*), and a common reference state defined by a known and easy-to-sample base distribution (*p*_0_). See Figure 1 for a qualitative illustration of the mapping between distributions, and SI Figure S1 for a description of the flow-based model architecture adopted in this work. This process naturally separates the task of computing free energy differences into two largely independent steps: obtaining the set of *K* reasonably accurate maps and obtaining free energy (FE) estimates via reweighting. Here, we focus primarily on the importance of the *second step* in achieving quantitatively accurate estimates of Δ*f*_*ij*_. We recognize that it may be possible that some types of ML maps may result in overlaps in phase space that may favor one type of free energy estimation method over another, but we assume for now that this training step can be separated from the estimation step.

The importance of reweighting is highlighted in the recent literature on the topic. For example, in refs (19 and 20) targeted BAR was used to obtain free energies of metastable states in small peptides and for drug-like molecules interacting with cucurbit[7]uril via flow-based PGMs. In refs (22 and 23) instead, flow-based maps were used in conjunction with a variant of targeted EXP to compute DFT free energies from distributions sampled with a classical force field in a diverse set of 22 drug-like molecules. Furthermore, ref (23) discusses how multiple separately trained maps sharing the same reference distribution can be used simultaneously in conjunction with MBAR.^13,14^

A common thread of refs (19,20,22,23) is the need to consider the effects of overfitting in the context of machine-learned mappings. For example, Ding and Zhang evaluated their DeepBAR estimators on separate data (i.e., validation data) absent from the training set.^20^ Meanwhile, Rizzi et al. contributed a dedicated discussion concluding that free energy estimates are more robust when evaluating EXP on validation data,^22^ and in ref (23), overfitting is avoided in a data-efficient manner by adopting a *one-epoch training policy*.

While these papers point at the need to carefully consider the reweighting approach, key differences in their intended applications and in the models adopted to develop invertible maps do not allow us to draw systematic conclusions on the accuracy and efficiency of different methods in relation to each other. In this paper, we set out to fill this gap and contribute to the current literature by systematically comparing the quantitative accuracy and convergence rates of all standard free energy estimators (AVMD, AVBG, EXPMD, EXPBG, BAR, and MBAR) defined in Section 2, in simple systems of increasing dimensionality. The comparisons are made in relation to ground truth separately obtained with the help of biased MD simulations using well-tempered metadynamics (WTmetaD).^24−27^ The details on MD and WTmetaD simulations are reported in Subsection 2.6 and in SI Figure S2. The key questions we aim to address are as follows:

How do the known free energy estimators compare in terms of their quantitative accuracy and convergence characteristics during model training?
How does training on a limited amount of data, inevitably leading to overfit models, impact the reweighting accuracy, and how can excessive overfitting be identified without a reference ground truth?
Can we devise any heuristics that can identify when free energy estimates are statistically consistent and robust without knowing the ground truth?

We tackle these questions by studying three model systems of increasing dimensionality, ranging from a three-dimensional model potential^28^ to the conformational ensembles of isolated organic molecules such as alanine dipeptide (AD) and ibuprofen. Throughout the current work, we rely on the cost-effectiveness and simplicity of maximum-likelihood-based training for fitting the *K* PGMs on locally ergodic data sets sampled separately from *K* nonoverlapping Boltzmann distributions via molecular dynamics (MD).

By systematically addressing this problem within well-defined confines, we draw conclusions and develop heuristics that are largely independent of the chosen remapping scheme, thus extending to the prolific literature of diverse flow-based machine learning models that have demonstrated significant potential in more complex systems.^18−23,29−32^

## 2 Theoretical Background: Free Energy Estimation via Reweighed Flow-Based Generative Models

In this section, we introduce the theoretical background on the reweighting and remapping methods that were used to compute free energy differences from PGMs, analyzed in the results section. We open this section with reweighting methods, that constitute the core of our analysis, and we note that such methods are independent of the *mapping* concept.

### 2.1 Reweighting Methods

Reweighting methods, including EXP and the BAR method, require estimates of ensemble averages on different ensembles. In the following, we adopt the typical shorthand notation ⟨*O*⟩_*p*_ to indicate the ensemble average of some generic state function *O*(***r***). The subscript *p* specifies that the average is taken with respect to probability distribution *p*(***r***). In practice, this is estimated on a data set of *N* ergodic samples {***r***} drawn from *p* in the following way:
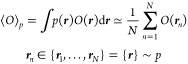
1

*EXP* is an example of one-directional reweighting. Given any normalized distribution *q* (say, a normalized Boltzmann distribution, or a flow-based PGM) and an exact equilibrium distribution in the canonical ensemble *p* = *Z*^–1^ exp (−*u*), and using the fact that 1 = ⟨*q*^–1^*p*⟩_*q*_ = ⟨*p*^–1^*q*⟩_*p*_, the dimensionless free energy *f* can be estimated as
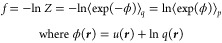
2

In the results section, estimates of *f* obtained from eq 2 are referred to with the prefix *EXP*. In particular we refer to –ln ⟨ exp (−ϕ)⟩_*q*_ as *EXPBG*, and ln⟨ exp (ϕ)⟩_*p*_ is referred to as *EXPMD*. The suffixes *BG* (Boltzmann Generator) or *MD* (Molecular Dynamics) indicate the origin of the samples used to estimate ensemble averages.

As discussed in the next section, focused on remapping 2.3, setting *q* to be a flow-based PGM with a known normalization constant implicitly introduces targeted functionality into eq 2, yielding a reweighted estimate of the absolute free energy *f*. In this manner, the EXPBG estimator was previously used^18,21,33^ to perform reweighting, where ϕ was defined as a *generalized work function*(21) or a *variational free energy*.^33^

*The two-state BAR method* is an example of two-directional reweighting that has been recently proposed as an efficient method to compute free energies from PGMs.^19,20^ Given a normalized mixture distribution *p*_M_ = (*N*_*p*_ + *N*_*q*_)^−1^(*N*_*p*_*p* + *N*_*q*_*q*) where *p* is an equilibrium distribution, *q* is a flow-based PGM, and *N*_*p*_ and *N*_*q*_ are the number of samples we may have available from *p* and *q*, respectively, for estimating the two ensemble averages below, one can write 1 = ⟨*p*_M_^–1^*p*⟩_*p*_M__. Using this equality, the reduced free energy *f* can be obtained by solving the following equation^14^:
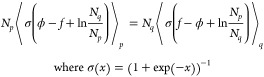
3

Estimates of *f* obtained by solving eq 3 are indicated as *BAR* in the results section. Similarly to eq 2, using ϕ in eq 3, as opposed to classical Δ*u*, implies remapping.

MBAR utilizes multidirectional reweighting, allowing it to jointly solve for free energy differences between an arbitrary *K* number of ensembles of interest, which are characterized by their respective probability distributions *p*_1_,..., *p*_*K*_. By rearranging 1 = ⟨*p*_M_^–1^*p*_*i*_⟩_*p*_M__, where *p*_M_ = (∑_*j*^′^ = 1_^*K*^*N*_*j*^′^_)^−1^ ∑_*j* = 1_^*K*^*N*_*j*_*p*_*j*_, it can be shown that the following well-known MBAR equation emerges^13,14^:
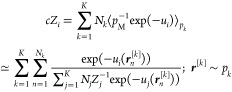
4

In practice, the solutions to eq 4 correspond to *cZ*_*i*_, where *c* is an arbitrary constant that does not affect the free energy differences. We note that in this scheme, we can collect samples from any number of physical or BG-generated samples that a given sampling scheme might suggest. In the limit of only two equilibrium distributions, eq 4 reduces to eq 3.^14^

In this work, the *pymbar* library^34^ was used to solve eqs 3 and 4, providing analytical error bars for free energy estimates, as highlighted in the results. The following sections overview the background behind remapping, defining the remapped potentials *u*_*k*→*i*_, and discussing the exact functional form of ln*q*, appearing in eqs 2 and 3, which was adopted in the current work.

### 2.2 Integration Limits

This work deals with metastable distributions of small-molecule conformers defined on a low dimensional collective-variable (CV) space. We wish to converge the free energy differences between the equilibrium ensembles corresponding to these conformers. We know that each conformer *i* is associated with a specific subset of the configurational space, and its free energy can be written as *f*_*i*_ = −ln ∫_*i*_ exp (−*u*)d***r***, where *u* = (*k*_B_*T*)^−1^*U* is a single global potential energy surface. However, we do not explicitly limit the architecture of the PGMs (SI Figure S1). to map precisely within the desired regions of the CV space. Ideally, the PGMs will learn these boundary conditions from the data, but the models are not guaranteed to ensure this for all samples they generate. In turn, throughout the current work, we define and implement potential energy surface of *i*th conformer *u*_*i*_ in the following way:
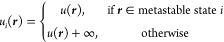
5

In eq 5, each configuration of the system ***r*** must be clustered using a fixed, deterministic clustering protocol specified a priori to obtain the cluster assignment of a metastable state. If the configuration does not belong to the correct metastable state, a significant energy penalty is added (e.g., 10^20^ in practice) to force the weight of that configuration to zero during reweighting. This effectively mimics the appropriate and consistent integration limits when taking ensemble averages. We observed that, for all cases considered in this work, this choice improves the rate of convergence of free energy estimates.

### 2.3 Remapping Normalized Distributions

Potential energy functions used in MD are typically defined on a 3*n*-dimensional orthonormal Cartesian coordinate system ***r*** = [*r*^1^,..., *r*^3*n*^], where *n* is the number of atoms in the system. An infinitesimal volume element in this coordinate system is d***r*** = ∏_*i* = 1_^3*n*^d*r*^*i*^. Provided with an invertible map  that can transform ***r*** into a different 3*n*-dimensional coordinate system ***z*** = _*r*→*z*_(***r***) = _*r*→*z*_(_*z*→*r*_(***z***)), which is not necessarily orthonormal, the volume element in the new coordinate system must be appropriately rescaled d***r*** = γ_*z*→*r*_(***z***)d***z***, setting , where *g*(***z***) is the metric tensor of the new coordinate system.^35^ Using the fact that the metric tensor of the original Cartesian coordinate system evaluates to an identity matrix at every point *g*(***r***) = *I*, it can be shown that γ can be written as it usually appears in normalizing flow literature, in terms of absolute determinant of either the forward or the backward Jacobian:
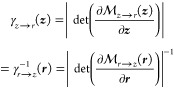
6

Equipped with eq 6, an infinitesimal mass element *p*_*r*_(***r***)d***r*** of a standard equilibrium distribution *p*_*r*_(***r***) = *Z*_*r*_^–1^ exp (−*u*_*r*_(***r***)) can be written as a function of ***z***:
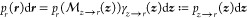
7

In turn, the remapped Boltzmann distribution *p*_*z*→*r*_ is defined in the following way, in which *u*_*z*→*r*_ is known as an *effective potential*.^22^
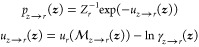
8

We now re-examine the distribution *q* appearing in eqs 2 and 3. If we introduce a new (e.g., uniform) distribution *p*_0_(***z***) = (∏_*i* = 1_^3*n*^∫_*a*_^*b*^d*z*^*i*^)^−1^ = *L*^–3*n*^, with *L* = *b*–*a*, then ln *p*_0_(***z***) = −3*n* ln *L*. Following a similar approach as in eq 7, the remapped version of *p*_0_ can be defined as *q*:
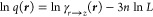
9

Utilizing this functional form of *q* implicitly couples remapping with EXP and BAR reweighting methods since we have direct access to the exact normalized likelihood values *q*(***r***). We can cheaply sample *q* by inverting samples from *p*_0_. All PGMs trained in the current paper were based on eq 7, with further details discussed in Section 2.4 and illustrated in SI Figure S1.

Similar to Rizzi et al.,^23^ in the current work, *targeted* MBAR was implemented by remapping through ***z*** (state 0) as an intermediate coordinate system. Unlike targeted EXP (eq 2) and BAR (eq 3), generalizing MBAR (eq 4) to the remapped version requires the definition of remapped potentials *u*_*k*→*i*_ and *u*_*k*→*j*_ (eq 8), which are substituted into eq 4 instead of *u*_*i*_ and *u*_*j*_, as previously derived in ref (23). Section 2.4 describes how distinct maps (_*i* ↔ 0_ ; *i* = 1,..., *K*) associated with *K* different (nonoverlapping) metastable states were trained to maximize their overlaps with *p*_0_ in the coordinate system associated with the unphysical state 0. This, in turn, allows all pairs of maps to be composed back to back in the following way:^23^
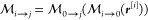
10

These maps (eq 10) were then used in eq 8 to obtain the corresponding remapped potentials, which were then employed within MBAR (eq 4). In this work, when performing MBAR, we used only samples obtained from MD and did not include samples from *p*_0_. We note that including samples from the common reference distribution *p*_0_ in MBAR is also an interesting possibility.

Maps are not necessarily accurate for *i* ≠ *j* since each was trained separately and is unlikely to overlap exactly in state _0_. As such, the accuracy of the targeted MBAR free energy estimates is expected to converge during training.

### 2.4 Normalizing Flow

Neural networks based on normalizing flow architectures (*flow-based*) are becoming increasingly popular for efficiently modeling flexible, multidimensional bijective functions , parametrized by a set of trainable parameters Θ (i.e.,  = ^[Θ]^). These functions are most often used in the context of eq 9, in which the base distribution *p*_0_ can be arbitrary.^18,21,36^ The goal of these methods is to minimize a probabilistic distance, usually Kullback–Leibler divergence (*D*_KL_) between the PGM *q* = *q*(***r***; ^[Θ]^) and the underlying known (or unknown) distribution of the data *p* = *p*(***r***). When *p* is a known Boltzmann distribution (up to the normalization constant), a PGM *q* sharing a sufficient overlap with *p* can be referred to as a BG.^18^ Many comprehensive reviews on normalizing flows have been published to date, for example.^37^ Finding the most appropriate normalizing flow architectures to model Boltzmann distributions in more general types of molecular systems is at the heart of ongoing work in the field. For example, recent developments make it possible to incorporate permutational invariance between multiple interacting molecules in a periodic box,^29^ with a means of coupling these degrees of freedom (DoF) to the shape of the box.^32^

The current paper deals with free energy estimation of metastable states of small molecules in a vacuum, specifically AD and ibuprofen. The underlying potential energy surfaces *u*_*i*_ and the corresponding equilibrium distributions of these states *p*_*i*_ are roto-translationally invariant on , without additional symmetries to be considered. SI Figure S1 illustrates the model structure of  adopted for all *q*_*i*_ = *q*(***r***; ^[Θ_*i*_]^) in this paper.^38−40^ This architecture relies on rational quadratic splines^41^ to transform marginal DoF of a mixed internal coordinate representation of the molecule.^42^ A 2D example of this is shown in 1. Correlations between DoF were treated using standard coupled flows architecture.^43^ All models *q*_*i*_ were individually trained by maximum likelihood, i.e., by minimizing only the *L*_ML_ error function with respect to the parameters Θ_*i*_:
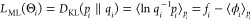
11

Due to *D*_KL_ being a nonsymmetric metric, a popular alternative regime of training has been previously described and recommended:^18^
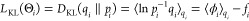
12

Although eq 12 was not used for training in the current work, training with either error function can hypothetically converge a PGM such that free energy can be estimated without reweighting:
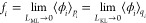
13

Assuming that an ideal mapping between *p*_0_ and *p*_*i*_ was learned, eq 13 shows that the absolute free energy of the *i*th metastable state *f*_*i*_ can be estimated by directly averaging its corresponding generalized work function ϕ_*i*_. In the analysis of the case studies discussed in the Results section, estimates of the *f*_*i*_ based on eq 13 are indicated with the prefix *AV* for averaging the work function. Estimates of *f*_*i*_ obtained by averaging ϕ over MD data (***r*** ∼ *p*) are indicated with AVMD. Estimates of *f*_*i*_ obtained by averaging ϕ over BG data (***r*** ∼ *q*) are instead indicated as AVBG. AVMD and AVBG have been previously mentioned.^18,19^ It is well-known that these estimators can only be accurate in the limit of an ideal mapping, i.e., for *L*_ML,KL_ → 0 (eqs 11 and 12).

### 2.5 Comparing Model Predictions with Ground Truth

To systematically assess the accuracy of free energy estimates obtained from PGMs, we consider three systems characterized by an increasing number of DoF: a 3D toy example^28^ (3 DoF), AD in a vacuum (60 DoF) at two separate temperatures 300 and 600 K, and ibuprofen in a vacuum (93 DoF) at 300 K.

When PGMs are trained on this data by maximum likelihood and then reweighted, the only new piece of information that all of the above-mentioned free energy estimations can provide is the configurational entropy difference (Δ*s* in units of *k*_B_) between pairs of metastable states. This quantity originates from the well-known equation of absolute configurational Helmholtz free energy *F*_*i*_ = ⟨*U*_*i*_⟩*_p_i__*–*TS*_*i*_, where *S*_*i*_ = ⟨−*k*_B_ ln *p*_*i*_⟩*_p_i__*. In temperature-reduced units *f*_*i*_ = (*k*_B_*T*)^−1^*F*_*i*_ = ⟨*u*_*i*_⟩*_p_i__*–*s*_*i*_, where the lower case entropy is *s*_*i*_ = ⟨−ln *p*_*i*_⟩*_p_i__*. This gives Δ*f*_*ij*_ = *C*_*ij*_ – Δ*s*_*ij*_, where *C*_*ij*_ = ⟨*u*_*j*_⟩*_p_j__* – ⟨*u*_*i*_⟩*_p_i__* and Δ*s*_*ij*_ = *s*_*j*_ – *s*_*i*_ = ⟨−ln *p*_*j*_⟩*_p_j__* – ⟨−ln *p*_*i*_⟩*_p_i__* = Δ*s*.

The differences between average potential energy *C*_*ij*_ are already available from the locally ergodic data sets used for training and validation. The accuracy of these quantities is independent of the quality of the reweighting scheme assessed. To concentrate on the latter, we focus our assessments on comparing Δ*s*_*ij*_ to ground truth Δ*s*_ij_^*^ while keeping the estimate of *C*_*ij*_ fixed.

The Δ*s*_*ij*_ estimates being tested are obtained from the separately fitted PGMs *q*_*i*_, trained by maximum likelihood on locally ergodic data sets {***r***}^[*i*]^ ∼ *p*_*i*_ sampled a priori via unbiased MD simulations, from separate metastable distributions *p*_*i*_.

### 2.6 MD Simulations

All simulations were carried out using the *OpenMM* Python library.^44^ The *ff99sb-ildn* force field was used for AD in a vacuum, and GAFF (from *prmtop* file^45^) was used for ibuprofen (IB) in a vacuum. No holonomic constraints were applied. All simulations were performed at a specified temperature *T* (Kelvin) using *LangevinIntegrator* with a friction coefficient of 1 ps^–1^ and a timestep of 2 fs. A trajectory frame was saved every 100 timesteps in unbiased simulations (u) and every 500 timesteps in biased simulations (b). Biasing was carried out in two-dimensional CV spaces of torsional angles using well-tempered metadynamics, in which a new repulsive Gaussian (*hill*) was deposited every 500 timesteps. The *hill* parameters can be found in the code.^45^ Specifically, the corresponding bandwidths were σ = 0.04 radians for AD at 300 and 600 K and σ = 0.04 radians for ibuprofen at 300 K. Initial height was *h*_0_ = 0.5 kJ/mol, and the bias temperature was Δ*T* = 3000 K. The convergence of free energy differences during those simulations is illustrated in SI Figure S2. In total, 1000, 500, 500, 400, 200, and 200 ns of MD data from respective simulations, IB300b, AD300b, AD600b, IB300u, AD300u, and AD600u, were eventually used. The AD300u and IB300u data sets were a concatenation of 10 simulations initialized from 10 different low-energy conformers in each metastable state. Each short simulation was saved after 10 ps of equilibration time to maintain a consistent averaged temperature. The AD600u data set was a single long trajectory. Global ergodicity of the unbiased data sets (AD300u, AD600u, and IB300u) was irrelevant in the current work since each PGM was intentionally trained on an equal number of training examples (conformers). Potential energies were evaluated efficiently using *bgflow/···/MultiContext* wrapper, applicable for any instance of *openmm.System*.^39^

### 2.7 PGM Training and Evaluation

All PGMs were trained using Adam optimizer fixed to a learning rate of 0.001, on batches size of 1000 random training examples drawn without replacement from the relevant training set in each training batch. For each metastable state in each system, the total amount of MD data allocated for training and evaluation (specified in results) was kept the same, with 50:50 training:validation split in each case. In the two molecules (AD and ibuprofen), random subsets of 10,000 conformers were used for evaluating the free energy estimators, drawn randomly without replacement from either training (T) or validation (V) data sets. In all cases, evaluations were done with a stride of 50 training batches during training, starting from the 50th batch. Notebooks implementing PGM training and evaluation and used to carry out all analyses are accessible at the URL https://github.com/E471r/RW_PGM_FE.^45^

## 3 Results and Discussion

### 3.1 Assessment of the Convergence of Free Energy Estimators in a 3D Toy-Model

**Figure 2:**
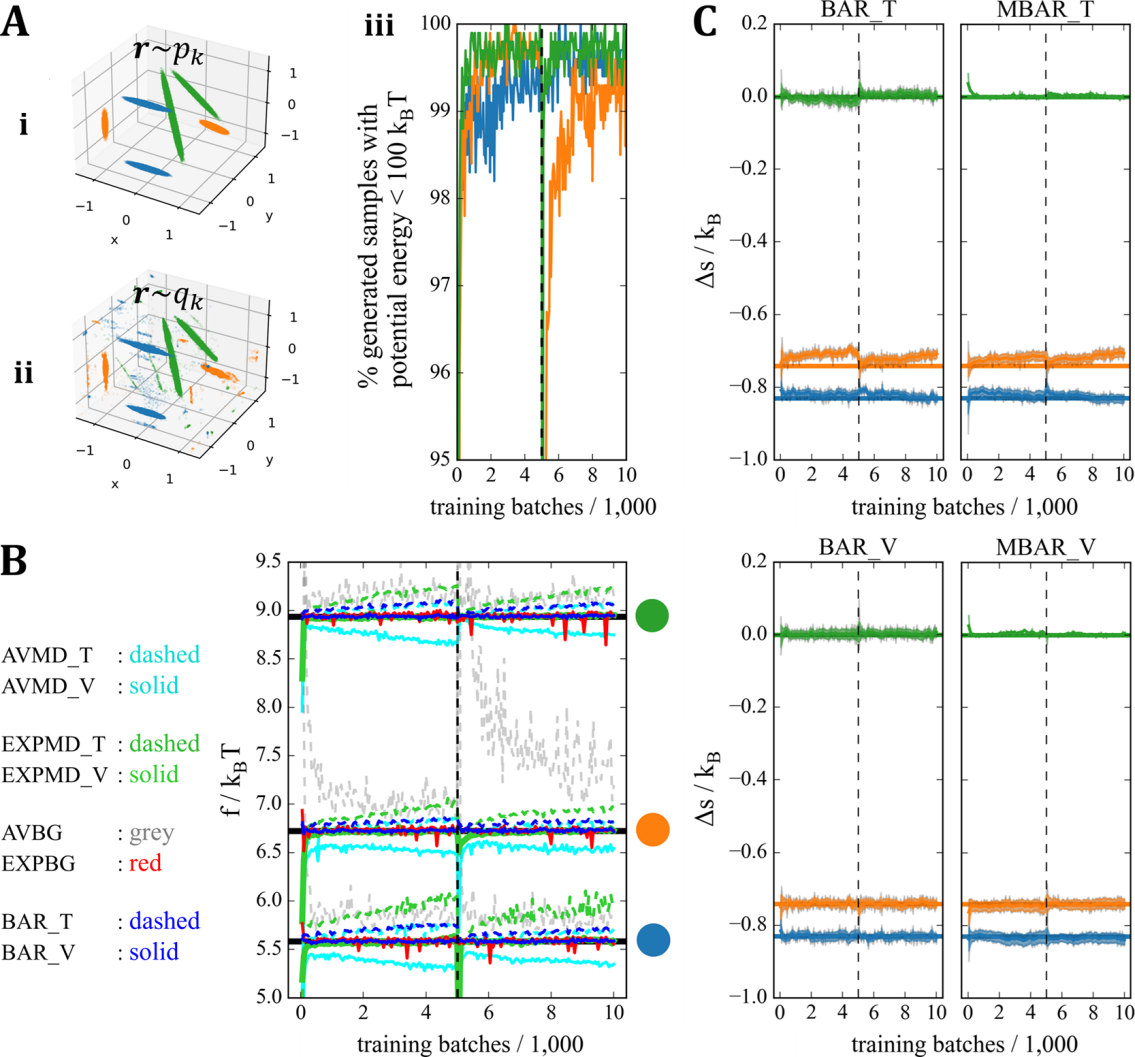
FE estimation in a toy example. The 3D potential^28^ discussed here has six 1-modal metastable states characterized by six narrow harmonic wells. These were arbitrarily split into three 2-modal metastable states (blue, orange, green) to mimic a general scenario where metastable ensembles of interest can be anharmonic. (A[i]) 10k locally ergodic training/validation data points from each 2-modal state are illustrated in the scatter plot. Only 1000 points from each state were used for training the separate models. Different sets of 1000 points per metastable state were used as validation data. The training/validation sets were swapped halfway through training, at which point the trainable parameters of the models were reset to their initial starting values. In each plot, this event is marked by a dashed vertical line appearing at 5,000 batches. The training was then resumed at the same learning rate for another 5,000 training steps. (A[ii]) Example of points sampled from three models after training. For every 50 training steps, 1000 samples were drawn from each model to evaluate the free energy estimators (B, C) and for plotting (A[iii]). (B) free energy estimators that yield absolute FEs are compared as a function of training progress. (C) Entropy differences based on free energy estimates provided by BAR and MBAR [using training (T) vs validation (V) data] are shown as a function of training progress. All horizontal lines indicate ground truth, readily available in 3D systems via quadrature.

The three-dimensional potential from ref (28) was chosen to initially test the six free energy estimators in a challenging regime of the very low amount of training (T) and validation (V) data, with both sets being locally ergodic within the colored metastable regions (Figure 2A[i]). The training was intentionally limited to small data sets to mimic a general scenario of possessing only a limited amount of MD data in relation to the accessible volume of the configurational space. As anticipated, maximum likelihood training on just 1000 samples manifested a noticeable overfitting behavior in each of the three PGMs during training. This, in turn, negatively affected all free energy estimators evaluated on the training data (Figure 2B,C). The estimators evaluated on the training data are labeled with a suffix _T. It can be seen that these free energy estimates increasingly diverged from the ground truth during training. On the other hand, the free energy estimates obtained by evaluating EXPMD, BAR, and MBAR on the validation data (unseen during model fitting), labeled with suffix _V, remained converged around the ground truth throughout training (Figure 2B,C). EXPBG, which only relies on samples from the models, also remained converged at the correct free energy values with low variance (Figure 2B). This favorable behavior persisted after the models were trained again with training and validation sets swapped (event marked by a dashed vertical line in Figure 2), indicating that the observed trends are independent of how the training and validation data are partitioned.

The heuristic of relying on validation data to obtain more accurate and robust free energy estimates in remapped distributions (based on flow-based PGMs trained on finite data) was previously recommended in ref (22). They described that systematic inaccuracies due to PGMs overfitting on the training data are expected to be less pronounced on average when calculating free energy differences on unseen validation data.^22^ The current results strongly support this heuristic, and it was interesting to verify this further in higher dimensional systems.

### 3.2 Assessment of the Convergence of Free Energy Estimators in AD in a Vacuum

**Figure 3:**
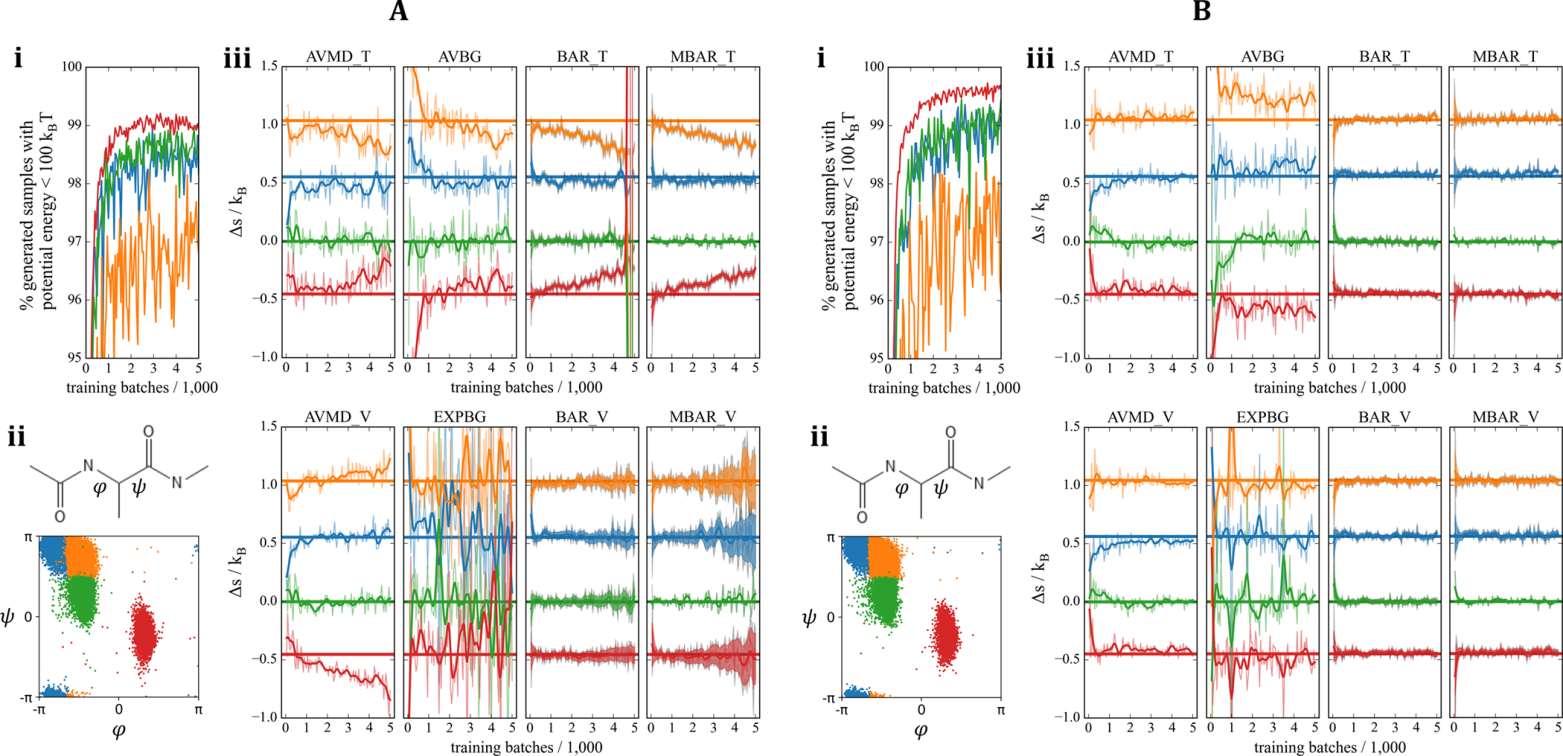
FE estimation of alanine dipeptide conformers in a vacuum at *T* = 300 K, using separate PGMs trained and evaluated on locally ergodic MD data sets at two different sample sizes. The states of interest (conformer ensembles) are illustrated using the four colors in the CV space spanned by torsional angles ϕ and ψ (A[ii], B[ii]). Specifically, the scatter plots show 2D projections of 10,000 samples drawn from each model at the end of training. (A) Low data regime (25,419 conformers for training (T) and 25,419 for validation (V); per state). (B) Higher data regime (60,647 conformers for training (T) and 60,647 for validation (V); per state). (A[i], B[i]) Percentage of conformers sampled from the models with reasonable potential energy (<100*k*_B_*T*) during training. (A[iii], B[iii]) Estimates of entropy difference between the four states using different methods, showing how overfitting has affected different estimators during training. The estimators that were generally too inaccurate (in the current case, only EXPMD) are not shown. The ground truth is illustrated by horizontal lines of corresponding colors and was obtained as described in II F (SI Figure S1) and in II E.

Repeating a similar procedure as in the toy example but for AD in a vacuum revealed similar trends. In Figure 3, separate PGMs were trained on four different conformer ensembles of AD (Figure 3A,B[ii]). In Figure 3A, the amount of MD data allocated for training was lower than in Figure 3B, while all other training and evaluation hyperparameters were kept fixed (2.7). In either case, the resulting four models could be considered qualitatively well-fitted, achieving and retaining good overlaps in potential energy shortly following the start of training (SI Figure S3). In turn, the differences seen between the performance of different free energy estimators in Figure 3A[iii] vs B[iii] are due to overfitting (i.e., due to PGMs being not sufficiently mass covering), and not due to under-fitting (lack of representational power of the maps). Similar to the trends already seen in the toy example, evaluating BAR and MBAR on the validation data (abbreviated as BAR_V and MBAR_V respectively) provided the most reliable free energy estimates, with the lowest variance compared to other methods, even during overfitting (Figure 3A[iii]). This was also observed in AD at *T* = 600 K (SI Figure S4). Importantly, the severity overfitting coincided with increasing analytical estimates of the error provided by BAR_V and MBAR_V. In turn, we hypothesize that keeping track of the analytical error bars provided by BAR/MBAR on the validation data is a useful measure of the quantitative accuracy of PGMs, which can be readily adopted as a heuristic in the absence of prior ground truth. To test this idea further, the following section looks at ibuprofen, a molecule with independent, anharmonic DoF compared to AD.

On a side note, in AD at *T* = 300 K, PGMs with the same architecture were also separately trained on the available biased MD data, obtained from the metadynamics calculations that were performed earlier to obtain ground-truth free energy differences, to check if reweighting via BAR would be able to recover the unbiased free energy differences (SI Figure S5). That result aligns well with previous conclusions about the robustness of BAR with respect to model accuracy.^20^ A possibility for training PGMs on biased data is, for instance, relevant when considering a broader utility of learned maps in umbrella sampling.

### 3.3 Assessment of the Convergence of Free Energy Estimators in Ibuprofen in a Vacuum

**Figure 4:**
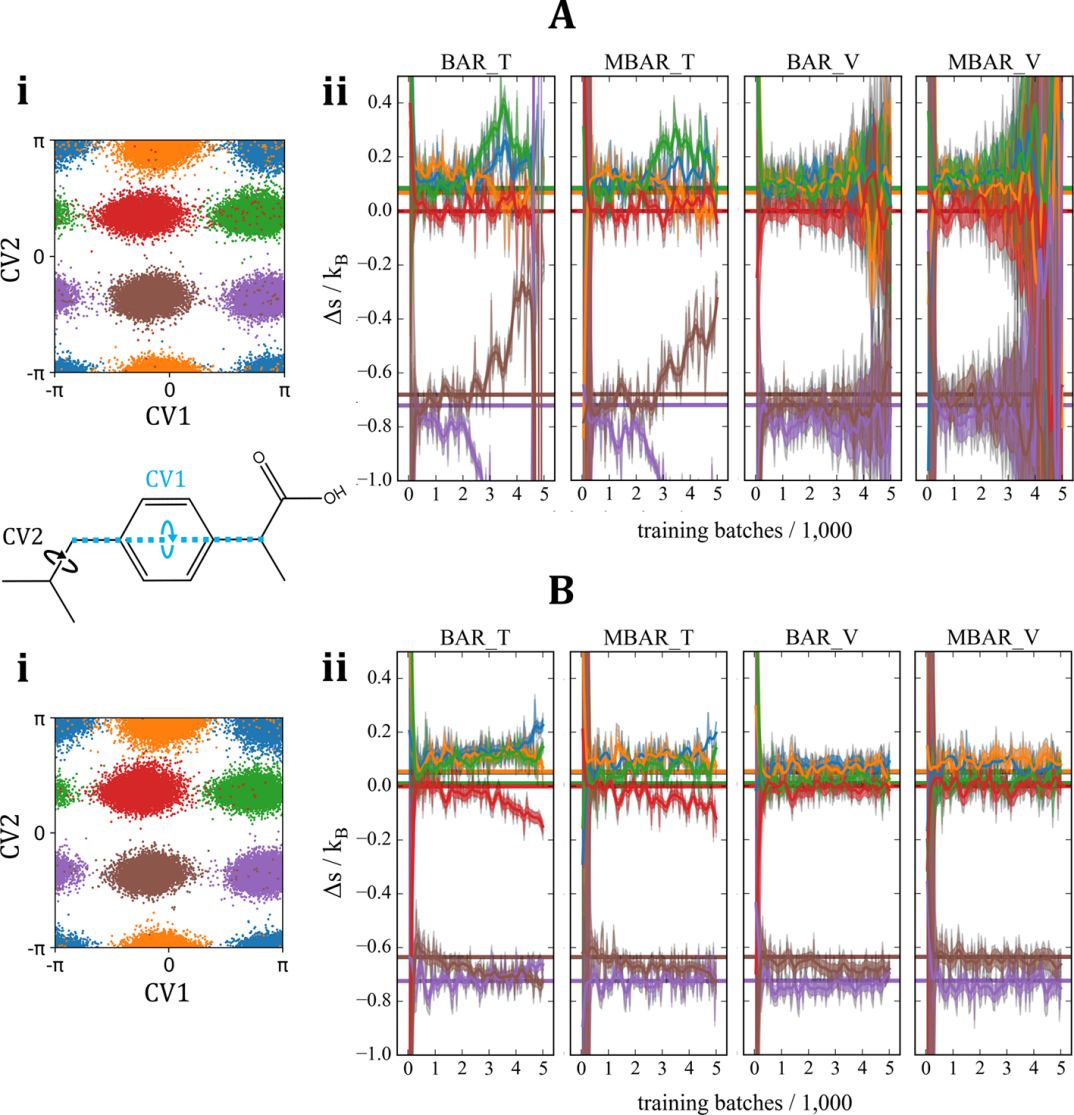
FE estimation of ibuprofen conformers in a vacuum at *T* = 300 K, using separate PGMs trained and evaluated on locally ergodic MD data sets at two different sample sizes, showing that moderate increases in training data can significantly reduce overfitting. The states of interest (conformer ensembles) are illustrated using the six colors in the CV space spanned by torsional angles CV1 and CV2 (A[i] and B[i]). Specifically, the scatter plots show 2D projections of 10,000 samples drawn from each model at the end of training. (A) Low data regime (30,590 conformers for training (T) and 30,590 for validation (V); per state). (B) Higher data regime (58,674 conformers for training (T) and 58,674 for validation (V); per state). (A[ii], B[ii]) Estimates of entropy difference between the six states using different methods. Only the estimators that were accurate on this scale are shown. The ground truth is illustrated by horizontal lines of corresponding colors and was obtained as described in II F (SI Figure S1) and in II E.

Repeating a similar procedure detailed in the AD case, but for ibuprofen in a vacuum at *T* = 300*K* and maintaining as many training hyperparameters similar as possible, reinforced the heuristics discussed in the previous sections (Figure 4). Specifically, Figure 4A shows all six PGMs significantly overfitting on the training data, especially after 2000 training batches. This behavior was characterized by diverging entropy estimates with misleadingly low error bars when evaluating BAR and MBAR on the training data. When evaluating BAR and MBAR on the validation data instead, the overfitting only caused increased error bars, retaining the ground truth within the error bars on average throughout further training (Figure 4A[ii]). In Figure 4B, the excessive overfitting was successfully remedied by approximately doubling the size of the training set. This measure prevented the error bars from increasing on the validation data, yielding reliable entropy estimates.

Our analysis of the overfitting behavior also underscores that tracking AVMD_V (i.e., ⟨ϕ_*i*_⟩*_p_i__* evaluated on validation data from *p*_*i*_) during training provides a useful measure of the quality of the model and thus of the corresponding free energy estimates. Using the model that maximizes AVMD_V was previously used in ref (20) as a heuristic for stopping the model training before the onset of overfitting. We verify here that this heuristics is valid also in all the case studies examined.

## 4 Conclusions

In this work, we focused on comparing and contrasting the accuracy of different reweighting methods applicable to the efficient and accurate estimation of free energy differences via machine-learned PGMs based on normalizing flows. We have systematically analyzed free energy differences between metastable states of systems of increasing complexity, from analytical potentials to organic molecules in a vacuum.

In this context, we have shown that while keeping the structure and complexity of the flow-based PGM fixed, the choice of reweighting method is crucial to obtain accurate free energy estimates. In particular, we have identified targeted BAR and MBAR as robust and data-efficient choices of free energy estimators.

Across all the scenarios tested, ground-truth free energy differences were consistently found within the error bars of the free energy estimates obtained by applying BAR or MBAR reweighting algorithms on validation data. We also observed that increasing the size of the locally ergodic training data sets allows for a systematic reduction of the analytical error bars, retaining free energy estimates consistent with the ground truth and avoiding overfitting. This observation underscores the value of using validation data when computing free energy from reweighted PGMs, systematically confirming observations that were episodically reported in prior literature.

In carrying out this analysis, we have noted that monitoring the quality of the free energy convergence on both validation and training data sets helped indicate the onset and severity of overfitting during training. Moreover, we noted that dividing the configuration space into metastable states, and using eq 5 to enforce this classification, has proven key to rapidly converge the training process.

In summary, we note that while we have limited our analysis to the reweighing of simple PGMs, trained to estimate free energy differences between metastable states of isolated molecules, our conclusions are derived generally independently of the choice of PGM and will also apply to more complex systems, provided a machine-learned mapping that guarantees sufficient overlap with the Boltzmann distribution of a target system. We envisage having a quantitative understanding of the impact of reweighting on the accuracy of PGM-based free energy calculations as an essential requirement for deploying efficient and accurate free energy calculation methods in complex systems such as molecular crystals and liquids.

## Supporting Information Available

Illustration of the flow-based model architecture used in the paper, convergence of the ground-truth free energy differences obtained via WTmetaD, potential energy overlaps obtained during training of AD at 300 K, free energy estimation of AD conformers at 600 K, free energy estimation and marginals of all the internal degrees of freedom for AD trained on data generated from WTmetaD, and energy overlap and marginals obtained from PGMs trained on separate ibuprofen metastable states (PDF)
