# Supplementary material for: Assessing the Accuracy and Efficiency of Free Energy Differences Obtained from Reweighted Flow-Based Probabilistic Generative Models
Source: J Chem Theory Comput. 2024 Jul 10;20(14):5913–22. doi: 10.1021/acs.jctc.4c00520 (PMC11270817; doi:10.1021/acs.jctc.4c00520)
Supplement: Supplementary file 1 — ct4c00520_si_001.pdf [file ct4c00520_si_001.pdf]

Supplementary Materials for: Assessing the accuracy and efficiency of free energy differences obtained from *reweighted* flow-based probabilistic generative models

Edgar Olehnovics,[1] Yifei Michelle Liu,[2] Nada Mehio,[3] Ahmad Y. Sheikh,[3] Michael R. Shirts,[4] and Matteo Salvalaglio[1]

[1] *Thomas Young Centre and Department of Chemical Engineering, University College London, London, WC1E 7JE, United Kingdom.*

[2] *Molecular Profiling and Drug Delivery, Research Development, AbbVie Bioresearch Center, Worcester, MA, 01605, USA*

[3] *Molecular Profiling and Drug Delivery, Research Development, AbbVie Inc, North Chicago, IL, 60064, USA*

[4] *University of Colorado Boulder, Boulder, CO, 80309, United States*

(*m.salvalaglio@ucl.ac.uk)

---

[1] Christina Gao, Joshua Isaacson, and Claudius Krause. i-flow: High-dimensional Integration and Sampling with Normalizing Flows. *Machine Learning: Science and Technology*, 1(4):045023, November 2020. arXiv:2001.05486 [hep-ph, physics:physics, stat].

[2] noegroup/bgflow, January 2024. original-date: 2021-04-15T15:37:40Z.

[3] Edgar Olehnovics. E471r/rw_pgm_fe, January 2024. original-date: 2024-01-29T12:20:29Z.

[4] Martín Abadi, Ashish Agarwal, Paul Barham, Eugene Brevdo, Zhifeng Chen, Craig Citro, Greg S. Corrado, Andy Davis, Jeffrey Dean, Matthieu Devin, Sanjay Ghemawat, Ian Goodfellow, Andrew Harp, Geoffrey Irving, Michael Isard, Yangqing Jia, Rafal Jozefowicz, Lukasz Kaiser, Manjunath Kudlur, Josh Levenberg, Dandelion Mané, Rajat Monga, Sherry Moore, Derek Murray, Chris Olah, Mike Schuster, Jonathon Shlens, Benoit Steiner, Ilya Sutskever, Kunal Talwar, Paul Tucker, Vincent Vanhoucke, Vijay Vasudevan, Fernanda Viégas, Oriol Vinyals, Pete Warden, Martin Wattenberg, Martin Wicke, Yuan Yu, and Xiaoqiang Zheng. TensorFlow: Large-scale machine learning on heterogeneous systems, 2015. Software available from tensorflow.org.

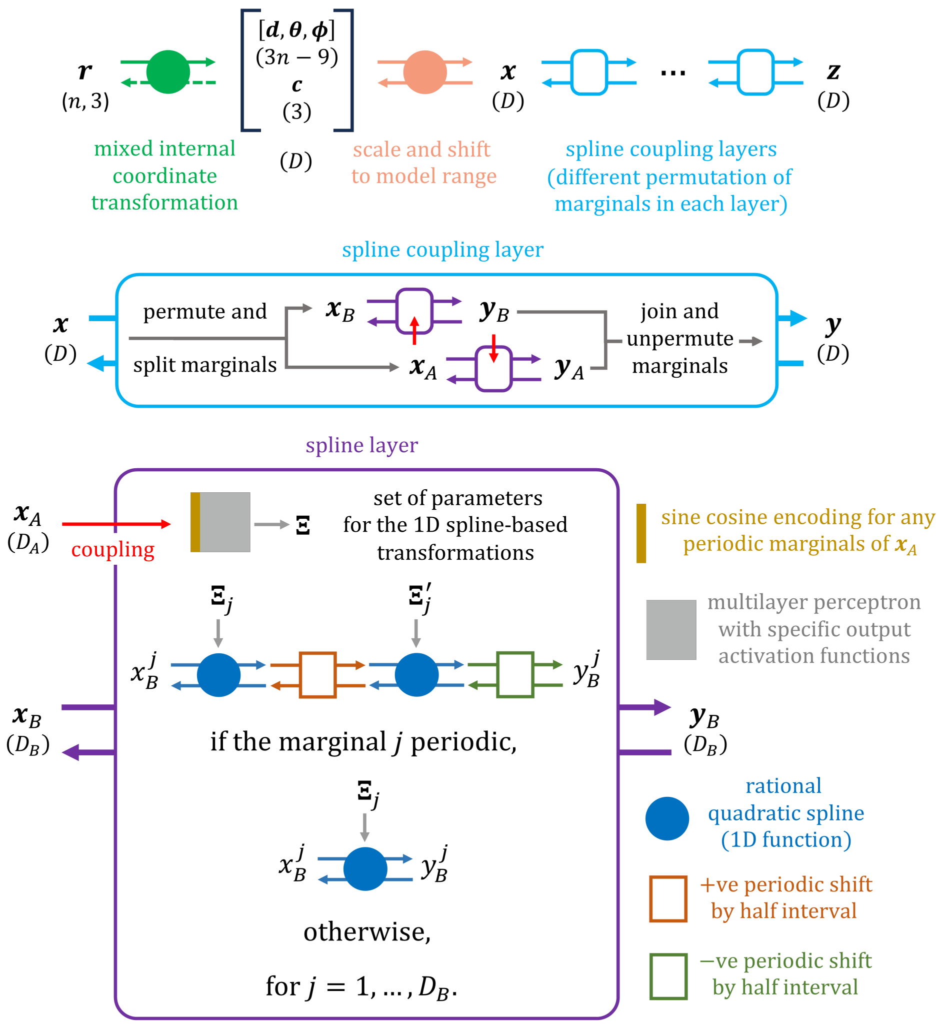

Figure S1: Illustration of the flow-based model architecture (map $\mathcal{M}$) used in the current work. For each vector-valued variable (bold), the number of marginal variables is shown in smooth brackets. In the case of molecules, the internal coordinate transformation begins with transforming 3D Cartesian coordinates of the molecule $\boldsymbol{r}$ (containing n atoms) into spherical polar coordinates for only $n-3$ atoms. The remaining three atoms are pre-aligned when pre-processing the training and validation MD data, so 6 out of 9 of their marginal Cartesian coordinates are constants. These constants are excluded from further transformations and only included when reconstructing the molecule. The three non-constant (non-singular) Cartesian coordinates remaining ($\boldsymbol{c}$) are shifted and rescaled to an arbitrary model range ($[-1,1]$ in the current case), together with the $3(n-3)$ spherical polar coordinates. Among the rescaled $D = 3(n-2)$ marginal variables contained in $\boldsymbol{x}$, $n-3$ marginal variables are periodic on the $[-1,1]$ interval (torsional angles $\boldsymbol{\phi}$), and the rest are non-periodic on this interval (bond distance $\boldsymbol{d}$, bond angles $\boldsymbol{\theta}$, and $\boldsymbol{c}$). All further transformations are trainable and carried out by spline-based bijectors (purple), arranged as shown in the spline coupling layer (cyan). Several independent spline coupling layers (cyan) are then stacked to transform between $\boldsymbol{x}$ and $\boldsymbol{z}$. The number of cyan layers used in the current work was 3 in the 3D toy system and 4 in the two molecules. The permutations of the marginals in each layer were set to a fixed random choice in each molecule, consistent in all metastable states. A more reliable non-random set of permutations in models like this was previously described in[1] but was not used here. The overall architecture we used contains no novel components and is largely inspired by publicly available libraries, such as *bg-flow*.[2] Some key components are cited either in the main text or the corresponding code.[3] The models were trained by gradient descent with respect to trainable parameters $\boldsymbol{\Theta}$ (contained within multilayered perceptrons; grey), using Adam optimizer in TensorFlow.[4]

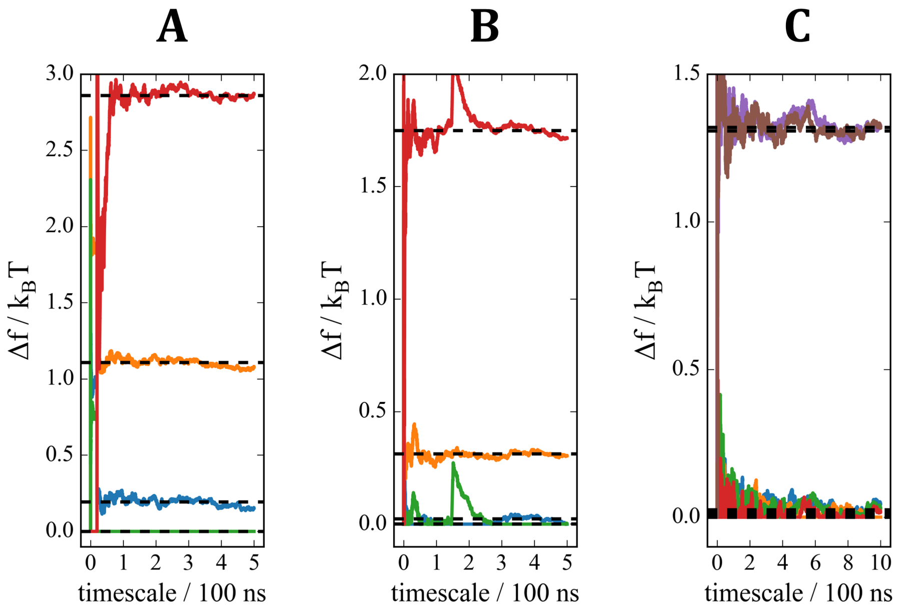

Figure S2: FE differences between conformers obtained via WTmetaD simulations. (A) Alanine dipeptide in vacuum at $T = 300K$. (B) Alanine dipeptide in vacuum at $T = 600K$. (C) Ibuprofen in vacuum at $T = 300K$. The colours represent metastable states in 2D CV spaces (discussed in the main text). Dashed, flat black lines represent the *ground truth* FE differences referred to in the main text.

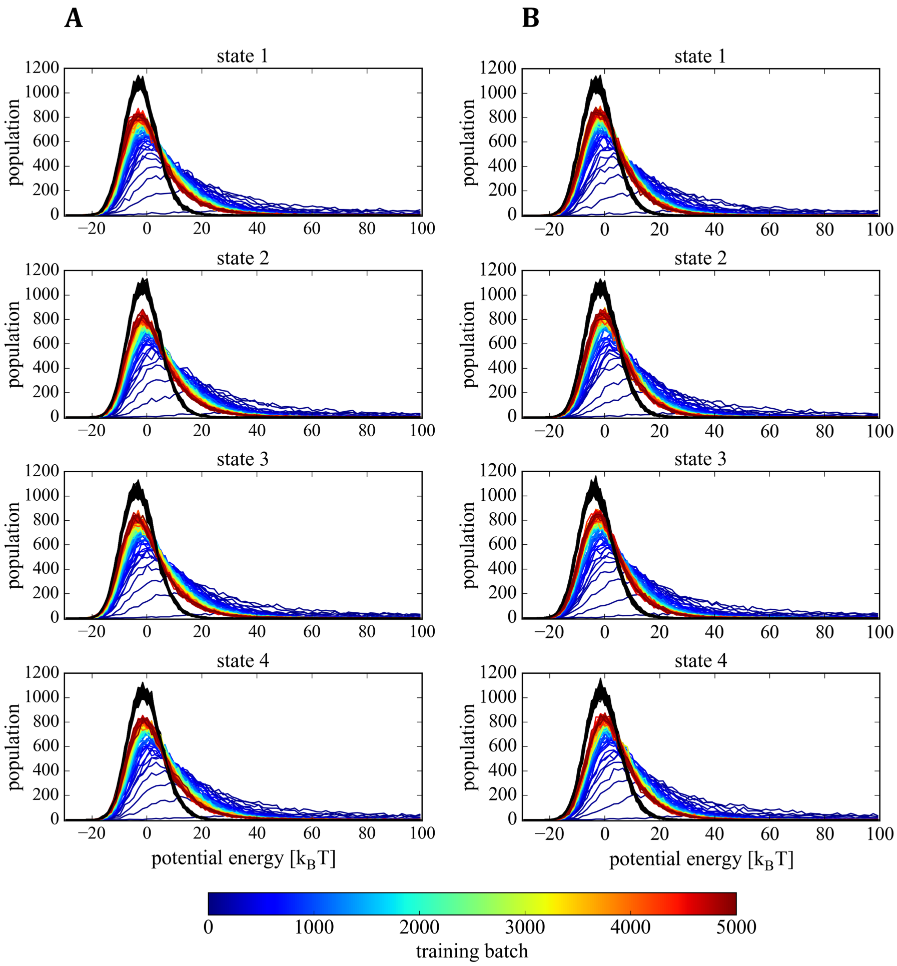

Figure S3: Potential energy overlaps during training on alanine dipeptide data in the $T = 300$ K case. This figure is linked to Fig.3 of the main text. Populations of potential energies of conformers sampled from the four models in low data regime (A) and high data regime (B) are shown. With a stride of 50 training steps (starting from 50) 10,000 conformers underlie each coloured histogram. The black histograms (two plotted every 50 training batches) are from 10,000 random conformers present in the relevant training and validation data. Each plot corresponds to the same conformers as used for BAR/MBAR reweighting at the corresponding training step.

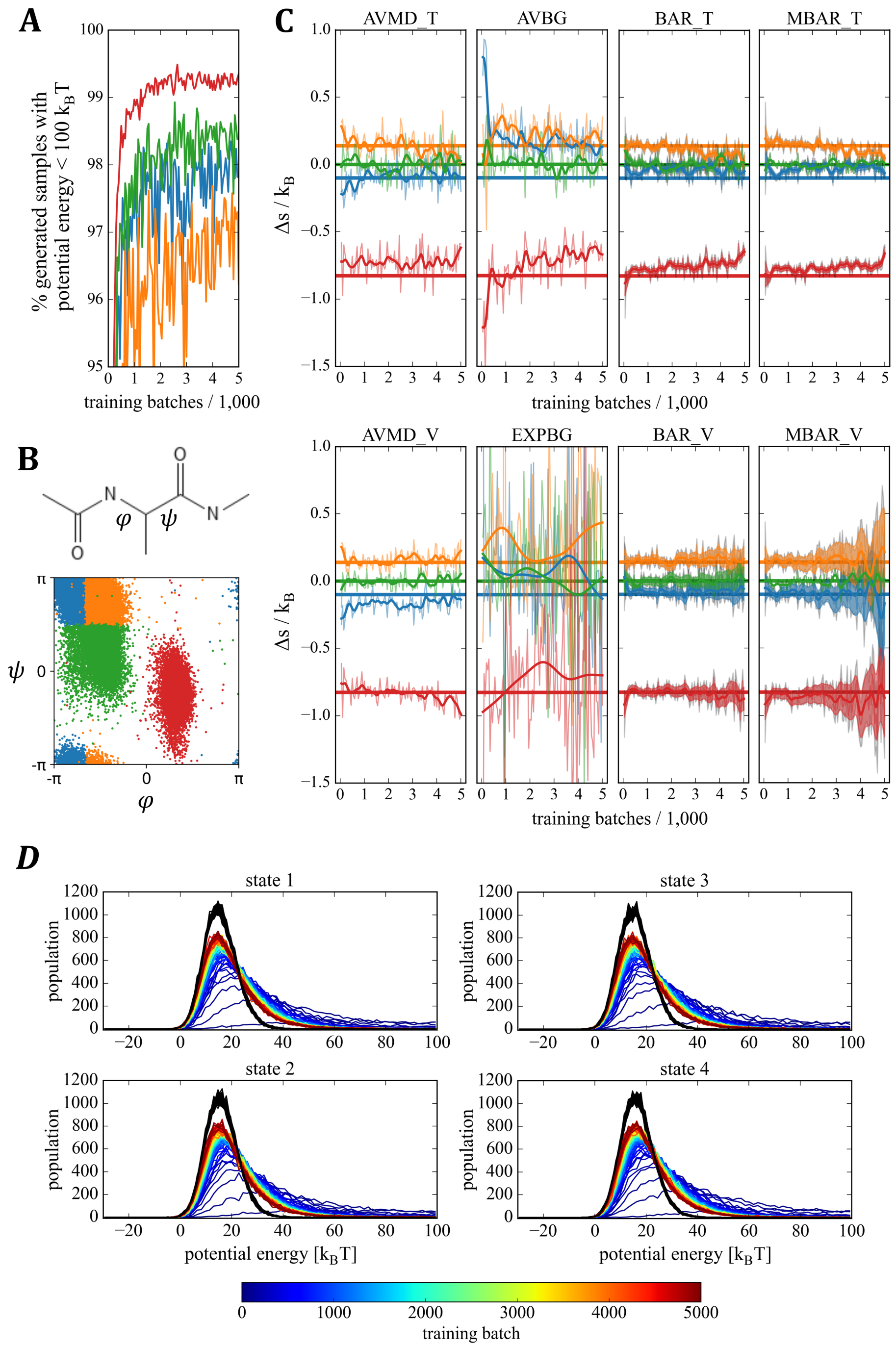

Figure S4: FE estimation of alanine dipeptide conformers in vacuum at $T = 600$ K on finite amount of MD data. This figure is directly analogous to Fig.3(A) of the main text, but here looking at a slightly different ensemble of alanine dipeptide (at double the temperature). (A-D) Low data regime (25,419 conformers for training (T) and 25,419 for validation (V); per state). (A) Percentage of conformers sampled from the models with reasonable potential energy ($< 100 k_B T$) during training. (B) Scatter plots of ϕ and ψ torsional angles in 10,000 samples drawn from each model at the end of training. (C) Entropy estimates during training using different methods. (D) Potential energy overlaps during training.

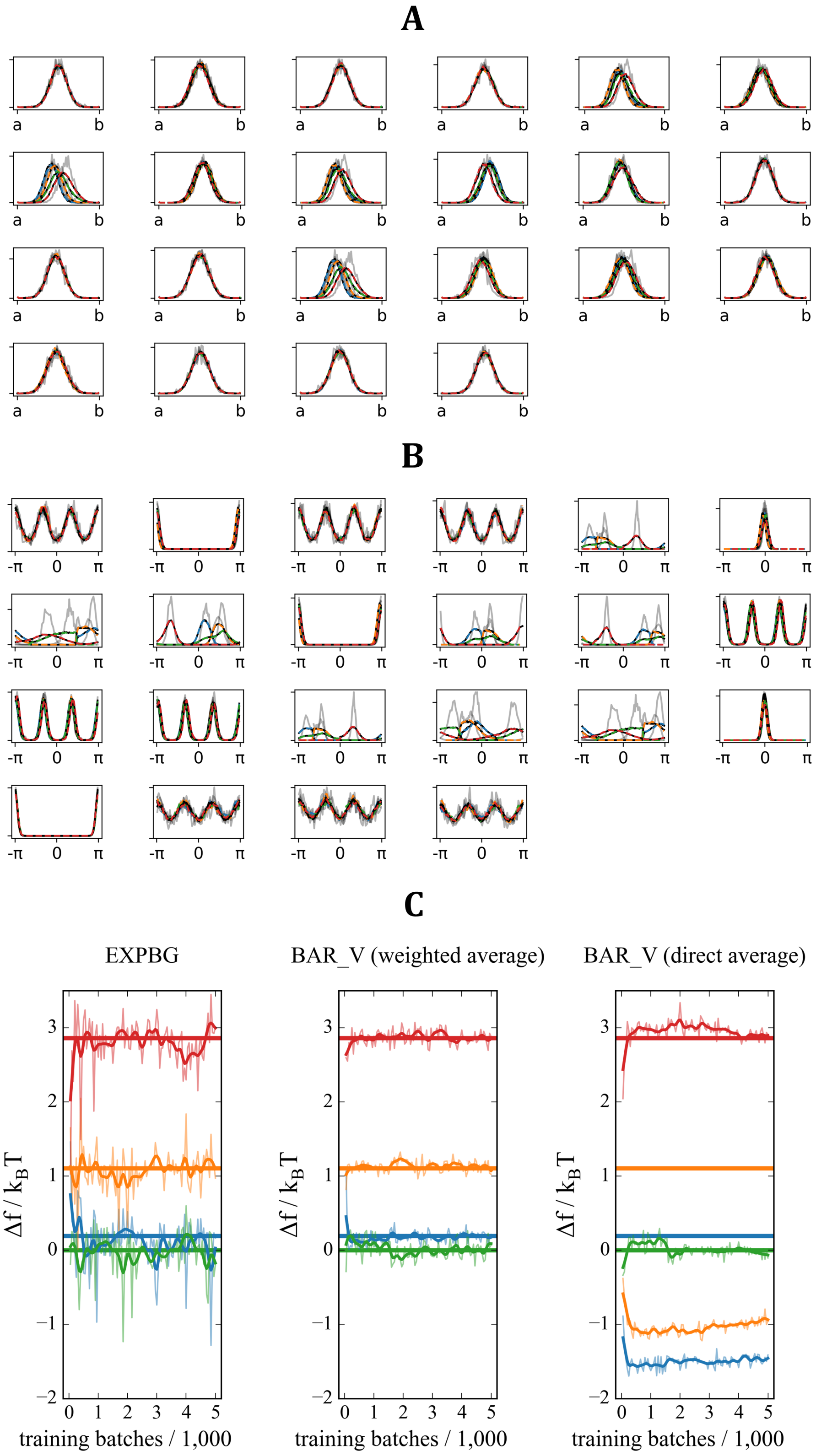

Figure S5: FE differences between conformers of alanine dipeptide obtained from models trained on biased data. (A) populations of bond angles in the MD data range $[a, b]$; $0 < a < b < \pi$. (B) populations of torsional angles. [black: (biased) training data, colour: samples from models, grey: Zwanzig reweighted training data] (C) Estimates of FE differences between the metastable states, compared to ground truth (flat lines).

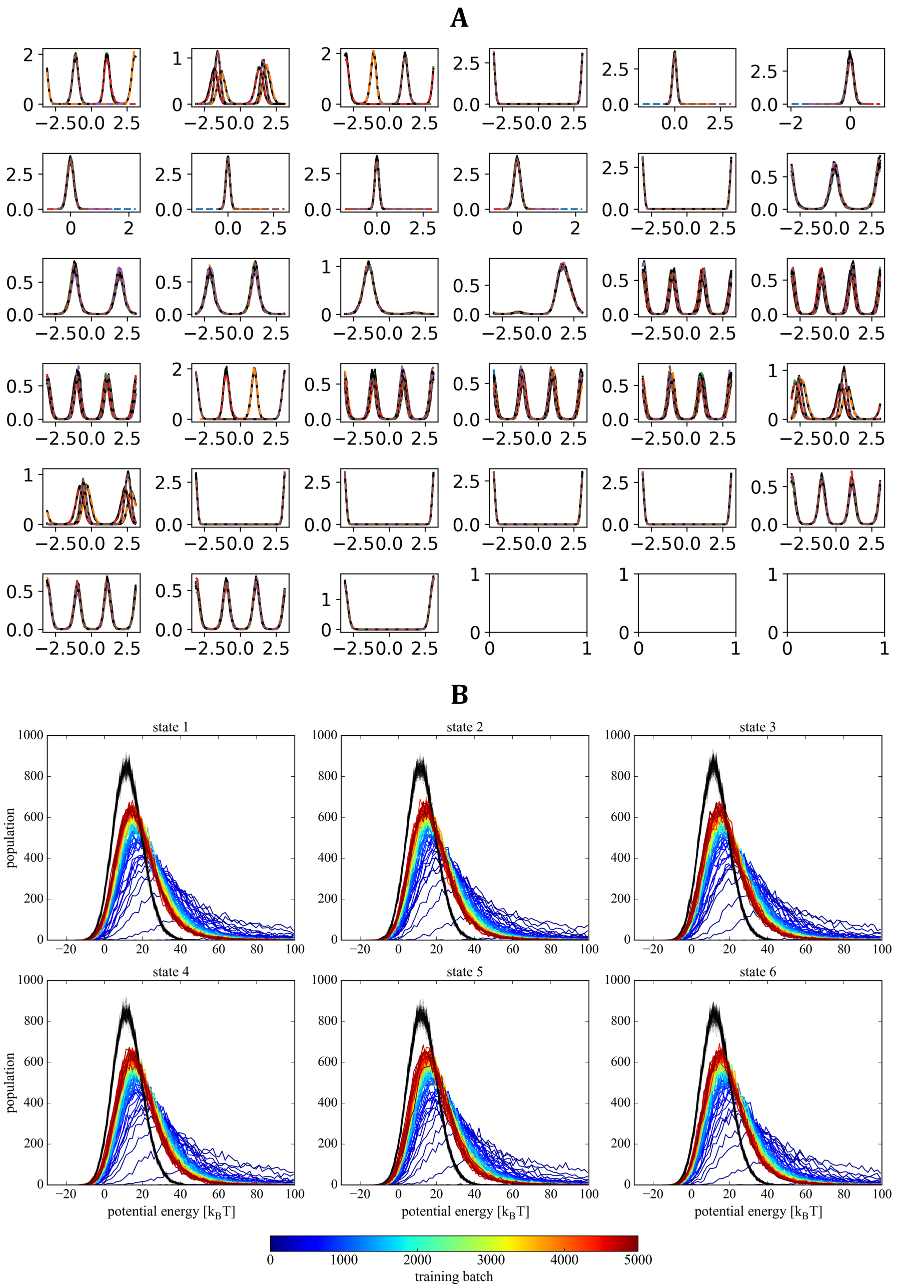

Figure S6: Qualitative look at PGMs trained on separate metastable states of ibuprofen. This figure is linked to Fig.4 (B) of the main text. (A) Marginal populations of ibuprofen torsional angles in conformers sampled from the six models. Black curves represent histograms on training data. Coloured curves represent histograms on the samples drawn from the six models. (B) Populations of potential energies of conformers sampled from the six models during training. Every 50 training steps (starting from 50), 10,000 conformers underlie each coloured histogram. The black histograms (two plotted every 50 training steps) are from 10,000 random conformers present in the training and validation data. Plots correspond to all of the conformers used for BAR reweighting during training.
